# Approximation to the global solution of generalized Zakharov equations in $\mathbf{R}^{2}$

**DOI:** 10.1186/s13660-018-1813-9

**Published:** 2018-08-22

**Authors:** Shujun You

**Affiliations:** School of Mathematical Sciences, Huaihua University, Huaihua, China

**Keywords:** Zakharov system, Generalized Zakharov equations, Approximation

## Abstract

We consider the initial value problem for the two-dimensional generalized Zakharov equations which model the propagation of Langmuir waves in plasmas. It is obtained that the solutions of the two-dimensional generalized Zakharov equations converge as $\alpha\to0$ to a solution of the Zakharov equations. Both weak and strong solutions are considered.

## Introduction

Zakharov derived a set of coupled nonlinear wave equations describing the interaction between high-frequency Langmuir waves and low-frequency ion-acoustic waves at the classical level [1]. The usual Zakharov system defined in space time $\mathbb{R}^{d+1}$ is given by

1 $$\begin{aligned}& iE_{t}+\Delta E=nE, \end{aligned}$$

2 $$\begin{aligned}& n_{tt}-\Delta n=\Delta|E|^{2}, \end{aligned}$$

where *E* is the envelope of the high-frequency electric field, and *n* is the plasma density measured from its equilibrium value.

Since 1972, this system has been the subject of a large number of studies [2–8]. Glangetas and Merle considered blow-up solutions of Zakharov equation in space dimension two. They proved various concentration properties of these solutions: existence, characterization of concentration mass, nonexistence of minimal concentration mass, and instability of periodic solutions [7, 8]. Ginibre, Tsutsumi, and Velo studied the local Cauchy problem in time for the Zakharov system governing Langmuir turbulence, with initial data $(u(0), n(0), \partial_{t} n(0))\in H^{k}\times H^{l} \times H^{l-1}$, in arbitrary space dimension. They proved that the Zakharov system is locally well-posed for a variety of values of $(k, l)$ [6].

The generalized Zakharov equation has also received the attention of many mathematicians [9–18]. Borhanifar et al. obtained generalized solitonary solutions and periodic solutions of the generalized Zakharov system and $(2 + 1)$-dimensional Nizhnik–Novikov–Veselov system by using the Exp-function method [9]. Guo et al. established local in time existence and uniqueness for a generalized Zakharov equation in the case of dimension $d=1, 2, 3$. Moreover, by using the conservation laws and the Brezis–Gallouet inequality, the solution can be extended globally in time in a two-dimensional case for small initial data. Besides, they also proved global existence of smooth solution in one spatial dimension without any small assumption for initial data [10]. Biswas et al. obtained the 1-soliton solution to Zakharov equation with power law and dual-power law nonlinearities. The He’s variational principle was used to carry out the integration of this equation [11]. Buhe and Bluman obtained several reductions and numerous new exact solutions of the generalized Zakharov equations by some subalgebras of symmetries [12]. Morris, Kara, and Biswas studied the Zakharov equation with power law nonlinearity. The traveling wave hypothesis was applied to obtain the 1-soliton solution of this equation. The multiplier method from Lie symmetries was subsequently utilized to obtain the conservation laws of the equations [13]. Linares and Pastor proved that the initial value problem for the two-dimensional modified Zakharov–Kuznetsov equation is locally well-posed for data in $H^{s}(\mathbb{R}^{2})$, $s>3/4$. Even though the critical space for this equation is $L^{2}(\mathbb{R}^{2})$, they proved that well-posedness is not possible in such a space. Global well-posedness and a sharp maximal function estimate were also established [14].

In [17], You studied the following generalized Zakharov system in space dimension two, and established the global existence for Cauchy problem.

3 $$\begin{aligned}& i E_{t}+\Delta E-nE+\alpha |E|^{p} E=0, \end{aligned}$$

4 $$\begin{aligned}& n_{tt}-\Delta n=\Delta|E|^{2}, \end{aligned}$$

5 $$\begin{aligned}& E|_{t=0}=E_{0}(x),\qquad n|_{t=0}=n_{0}(x), \qquad n_{t}|_{t=0}=n_{1}(x). \end{aligned}$$

We are interested in this paper in the asymptotic behavior of system (3)–(4) when *α* goes to zero. We regard equations (3)–(4) as the Langmuir turbulence parameterized by *α* ($|\alpha|<1$). One expects that the sequence of system (3)–(4) converges to the Zakharov equations (1)–(2). Actually, our goal is to show that the solutions $(E^{\alpha}, n^{\alpha})$ to (3)–(4) tend to $(E, n)$ when *α* goes to 0, where $(E, n)$ is the solution to (1)–(2) with (5).

The obtained results may be useful for better understanding the long wave Langmuir turbulence in plasma. Now we state the main results of the paper.

### Theorem 1.1

*Assume that* $E_{0}\in H^{1}(\mathbb{R}^{2})$, $n_{0}\in L^{2}(\mathbb{R}^{2})$, $n_{1}\in H^{-1}(\mathbb{R}^{2})$, $0< p\leq2$, $|\alpha|<1$, *and* $\| E_{0}(x)\|_{L^{2}}$ *small*. *Then*, *as* *α* *goes to zero*, $(E^{\alpha}, n^{\alpha})$ *converges to* $(E, n)$ *in* $L^{\infty}(\mathbb{R}_{+}, H^{1}(\mathbb{R}^{2}))\times L^{\infty }(\mathbb{R}_{+}, L^{2}(\mathbb{R}^{2}))$ *weakly star*.

### Theorem 1.2

*Suppose that* $E_{0}\in H^{m+2}(\mathbb{R}^{2})$, $n_{0}\in H^{m+1}(\mathbb {R}^{2})$, $n_{1}\in H^{m}(\mathbb{R}^{2})$ ($m\geq1$) , $0< p\leq2$, $|\alpha |<1$, *and* $\|E_{0}(x)\|_{L^{2}}$ *small*. *Then*

$$\begin{aligned} & \bigl\Vert F^{\alpha}\bigr\Vert _{H^{m}(\mathbb{R}^{2})}= \bigl\Vert E^{\alpha}-E \bigr\Vert _{H^{m}(\mathbb{R}^{2})}\rightarrow0\quad (\alpha \rightarrow0), \\ & \bigl\Vert u^{\alpha}\bigr\Vert _{H^{m-1}(\mathbb{R}^{2})}= \bigl\Vert n^{\alpha}-n \bigr\Vert _{H^{m-1}(\mathbb{R}^{2})}\rightarrow0 \quad (\alpha \rightarrow0). \end{aligned}$$

For the sake of convenience of the following contexts, we set some notations. For $1 \leq q \leq\infty$, we denote by $L^{q}(\mathbb{R}^{2})$ the space of all *q* times integrable functions in $\mathbb{R}^{2}$ equipped with the norm $\|\cdot\|_{L^{q}(\mathbb{R}^{2})}$ or simply $\|\cdot\|_{L^{q}}$ and by $H^{s}(\mathbb{R}^{2})$ the Sobolev space with the norm $\|\cdot\| _{H^{s,2}(\mathbb{R}^{2})}$. Let $(f,g)=\int_{\mathbb{R}^{2}}f(x)\cdot\overline{g(x)} \,dx$, where $\overline{g(x)}$ denotes the complex conjugate function of $g(x)$.

The paper is organized as follows. In Sect. 2, we establish a weak convergence result. In Sect. 3, we state the strong convergence results.

## Weak convergence results

In this section, we shall prove Theorem 1.1. We introduce *φ* and transform (3)–(4) with (5) into the following form:

6 $$\begin{aligned} &iE_{t}^{\alpha}+ \Delta E^{\alpha}-n^{\alpha}E^{\alpha}+\alpha \bigl\vert E^{\alpha}\bigr\vert ^{p} E^{\alpha}=0, \end{aligned}$$

7 $$\begin{aligned} &n_{t}^{\alpha}-\Delta\varphi^{\alpha}=0, \end{aligned}$$

8 $$\begin{aligned} &\varphi_{t}^{\alpha}-n^{\alpha}= \bigl\vert E^{\alpha}\bigr\vert ^{2}, \end{aligned}$$

with the initial data

9 $$ E^{\alpha}|_{t=0}=E_{0}(x),\qquad n^{\alpha}|_{t=0}=n_{0}(x),\qquad \varphi ^{\alpha}|_{t=0}=\varphi_{0}(x), $$

where $\varphi_{0} $ satisfies $\Delta\varphi_{0}=n_{1}$.

Take the inner product of (6) and $E^{\alpha}$, and take the inner product of (6) and $E^{\alpha}_{t}$. Then we can obtain the following invariants:

10 $$\begin{aligned}& \bigl\Vert E^{\alpha}\bigr\Vert ^{2}_{L^{2}(\mathbb{R}^{2})}= \Vert E_{0} \Vert ^{2}_{L^{2}(\mathbb {R}^{2})}, \end{aligned}$$

11 $$\begin{aligned}& \begin{aligned}[b] \mathscr{H}(t)&:= \bigl\Vert \nabla E^{\alpha}\bigr\Vert ^{2}_{L^{2}(\mathbb {R}^{2})}+\frac{1}{2} \bigl\Vert \nabla\varphi^{\alpha}\bigr\Vert ^{2}_{L^{2}(\mathbb{R}^{2})}+ \frac {1}{2} \bigl\Vert n^{\alpha}\bigr\Vert ^{2}_{L^{2}(\mathbb{R}^{2})} \\ &\quad {} + \int_{\mathbb{R}^{2}} n^{\alpha}\bigl\vert E^{\alpha}\bigr\vert ^{2}\,dx-\frac{2\alpha}{p+2} \int _{\mathbb{R}^{2}} \bigl\vert E^{\alpha}\bigr\vert ^{p+2}\,dx=\mathscr{H}(0). \end{aligned} \end{aligned}$$

In this section, we consider the initial data satisfying rather few regularity conditions. More precisely, we assume that $E_{0}$, $n_{0}$, $n_{1}$ lie in $H^{1}(\mathbb{R}^{2})$, $L^{2}(\mathbb{R}^{2})$, $H^{-1}(\mathbb{R}^{2})$, respectively. According to the Galerkin method, there exists the weak global solution

$$ E^{\alpha}\in L^{\infty}\bigl(\mathbb{R}_{+}, H^{1}\bigl( \mathbb{R}^{2}\bigr)\bigr),\qquad n^{\alpha}\in L^{\infty} \bigl(\mathbb{R}_{+}, L^{2}\bigl(\mathbb{R}^{2}\bigr)\bigr). $$

The proof of Theorem 1.1 needs two lemmas recalled in [19].

### Lemma 2.1

*Let* $B_{0}$, *B*, $B_{1}$ *be three reflexive Banach spaces and assume that the embedding* $B_{0}\rightarrow B$ *is compact*. *Let*

$$ W= \biggl\{ V\in L^{p_{0}}\bigl((0, T); B_{0}\bigr), \frac{\partial V}{\partial t}\in L^{p_{1}}\bigl((0, T); B_{1}\bigr) \biggr\} , \quad T< \infty, 1< p_{0},p_{1}< \infty. $$

*W* *is a Banach space with the norm*

$$ \|V\|_{W}=\|V\|_{L^{p_{0}}((0, T); B_{0})}+\|V_{t}\|_{L^{p_{1}}((0, T); B_{1})}. $$

*Then the embedding* $W\rightarrow L^{p_{0}}((0, T); B)$ *is compact*.

### Lemma 2.2

*Let* Ω *be an open set of* $\mathbb{R}^{n}$, *and let* $g,g_{\varepsilon}\in L^{p}(\mathbb{R}^{n})$, $1< p<\infty$, *such that*

$$ g_{\varepsilon}\rightarrow g \quad \textit{a.e. in } \Omega \quad \textit{and} \quad \| g_{\varepsilon}\|_{L^{p}(\Omega)}\leq C. $$

*Then* $g_{\varepsilon}\rightarrow g$ *weakly in* $L^{p}(\Omega)$.

Now, with these lemmas, we are able to prove Theorem 1.1.

### Proof

By Hölder’s inequality, Young’s inequality, and the Gagliardo–Nirenberg inequality, there holds

$$\begin{aligned} \biggl\vert \int_{\mathbb{R}^{2}}n^{\alpha}\bigl\vert E^{\alpha}\bigr\vert ^{2}\,dx \biggr\vert &\leq \bigl\Vert n^{\alpha}\bigr\Vert _{L^{2}} \bigl\Vert E^{\alpha}\bigr\Vert ^{2}_{L^{4}}\leq\frac{1}{4} \bigl\Vert n^{\alpha}\bigr\Vert ^{2}_{L^{2}}+ \bigl\Vert E^{\alpha}\bigr\Vert ^{4}_{L^{4}} \\ &\leq\frac{1}{4} \bigl\Vert n^{\alpha}\bigr\Vert ^{2}_{L^{2}}+C \bigl\Vert \nabla E^{\alpha}\bigr\Vert ^{2}_{L^{2}} \bigl\Vert E^{\alpha}\bigr\Vert ^{2}_{L^{2}}. \end{aligned}$$

Using the Gagliardo–Nirenberg inequality and noting $|\alpha|<1$, we write

$$ \frac{2|\alpha|}{p+2} \bigl\Vert E^{\alpha}\bigr\Vert ^{p+2}_{L^{p+2}}\leq\frac{2}{p+2} \bigl\Vert E^{\alpha}\bigr\Vert ^{p+2}_{L^{p+2}}\leq C \bigl\Vert \nabla E^{\alpha}\bigr\Vert ^{p}_{L^{2}} \bigl\Vert E^{\alpha}\bigr\Vert ^{2}_{L^{2}}. $$

Note that $0< p\leq2$ and $\|E_{0}\|_{L^{2}}$ small, relations (10) and (11) imply that the quantities

$$ \bigl\Vert E^{\alpha}\bigr\Vert _{L^{\infty}(\mathbb{R}_{+}, H^{1}(\mathbb{R}^{2}))},\qquad \bigl\Vert n^{\alpha}\bigr\Vert _{L^{\infty}(\mathbb{R}_{+}, L^{2}(\mathbb{R}^{2}))}, \qquad \bigl\Vert \varphi^{\alpha}\bigr\Vert _{L^{\infty}(\mathbb{R}_{+}, H^{1}(\mathbb{R}^{2}))} $$

are bounded uniformly in *α*. Therefore, some subsequence of $(E^{\alpha}, n^{\alpha}, \varphi^{\alpha})$, also labeled by *α*, has a weak limit $(E, n, \varphi)$. More precisely

12 $$\begin{aligned} &E^{\alpha}\to E \quad \mbox{in } L^{\infty}\bigl( \mathbb{R}_{+}, H^{1}\bigl(\mathbb{R}^{2}\bigr)\bigr) \mbox{ weakly star}, \end{aligned}$$

13 $$\begin{aligned} &n^{\alpha}\to n \quad \mbox{in } L^{\infty}\bigl( \mathbb{R}_{+}, L^{2}\bigl(\mathbb{R}^{2}\bigr)\bigr) \mbox{ weakly star}, \end{aligned}$$

14 $$\begin{aligned} &\varphi^{\alpha}\to\varphi\quad \mbox{in } L^{\infty} \bigl(\mathbb {R}_{+}, H^{1}\bigl(\mathbb{R}^{2}\bigr)\bigr) \mbox{ weakly star}. \end{aligned}$$

Moreover, let us note that the following maps are continuous:

$$ H^{1}\bigl(\mathbb{R}^{2}\bigr)\rightarrow L^{4} \bigl(\mathbb{R}^{2}\bigr) \quad \mbox{and}\quad H^{1}\bigl( \mathbb{R}^{2}\bigr)\times L^{2}\bigl(\mathbb{R}^{2} \bigr)\rightarrow H^{-1}\bigl(\mathbb{R}^{2}\bigr). $$

It then follows from (12) and (13) that the quantities

$$\begin{aligned} & \bigl\Vert \Delta E^{\alpha}\bigr\Vert _{L^{\infty}(\mathbb{R}_{+}, H^{-1}(\mathbb{R}^{2}))},\qquad \bigl\Vert n^{\alpha}E^{\alpha}\bigr\Vert _{L^{\infty}(\mathbb{R}_{+}, H^{-1}(\mathbb{R}^{2}))},\qquad \bigl\Vert \bigl\vert E^{\alpha}\bigr\vert ^{2} \bigr\Vert _{L^{\infty}(\mathbb{R}_{+}, L^{2}(\mathbb {R}^{2}))}, \\ & \bigl\Vert \Delta\varphi^{\alpha}\bigr\Vert _{L^{\infty}(\mathbb{R}_{+}, H^{-1}(\mathbb {R}^{2}))},\qquad \bigl\Vert \alpha \bigl\vert E^{\alpha}\bigr\vert ^{p}E^{\alpha}\bigr\Vert _{L^{\infty}(\mathbb {R}_{+}, L^{2}(\mathbb{R}^{2}))} \end{aligned}$$

are bounded uniformly in *α*. So it can be assumed that

15 $$\begin{aligned} & n^{\alpha}E^{\alpha}\mbox{ has a weak limit } z \mbox{ in } L^{\infty}\bigl(\mathbb{R}_{+}, H^{-1}\bigl( \mathbb{R}^{2}\bigr)\bigr), \end{aligned}$$

16 $$\begin{aligned} & \bigl\vert E^{\alpha}\bigr\vert ^{2} \mbox{ has a weak limit } w \mbox{ in } L^{\infty}\bigl(\mathbb{R}_{+}, L^{2}\bigl(\mathbb{R}^{2}\bigr)\bigr), \end{aligned}$$

17 $$\begin{aligned} & \alpha \bigl\vert E^{\alpha}\bigr\vert ^{p}E^{\alpha}\mbox{ has a weak limit } v \mbox{ in } L^{\infty}\bigl(\mathbb{R}_{+}, L^{2}\bigl(\mathbb{R}^{2} \bigr)\bigr), \end{aligned}$$

and

18 $$\begin{aligned} &\Delta E^{\alpha}\rightarrow\Delta E \quad \mbox{in } L^{\infty }\bigl(\mathbb{R}_{+}, H^{-1}\bigl(\mathbb{R}^{2} \bigr)\bigr) \mbox{ weakly star}, \end{aligned}$$

19 $$\begin{aligned} &\Delta\varphi^{\alpha}\rightarrow\Delta\varphi\quad \mbox{in } L^{\infty}\bigl(\mathbb{R}_{+}, H^{-1}\bigl( \mathbb{R}^{2}\bigr)\bigr) \mbox{ weakly star}. \end{aligned}$$

Finally, taking into account (12)–(19), Eqs. (6)–(8) imply that

20 $$\begin{aligned} & E^{\alpha}_{t}\rightarrow E_{t}\quad \mbox{in } L^{\infty}\bigl(\mathbb {R}_{+}, H^{-1}\bigl( \mathbb{R}^{2}\bigr)\bigr) \mbox{ weakly star}, \end{aligned}$$

21 $$\begin{aligned} & n^{\alpha}_{t}\rightarrow n_{t} \quad \mbox{in } L^{\infty}\bigl(\mathbb {R}_{+}, H^{-1}\bigl( \mathbb{R}^{2}\bigr)\bigr) \mbox{ weakly star}, \end{aligned}$$

22 $$\begin{aligned} & \varphi^{\alpha}_{t}\rightarrow \varphi_{t} \quad \mbox{in } L^{\infty}\bigl(\mathbb{R}_{+}, L^{2}\bigl(\mathbb{R}^{2}\bigr)\bigr) \mbox{ weakly star}. \end{aligned}$$

Using the above results, the proof of Theorem 1.1 will be complete if we establish that

$$ z=nE,\qquad w=|E|^{2},\quad \mbox{and}\quad v=0. $$

Let Ω be any bounded subdomain of $\mathbb{R}^{2}$, *ψ* be some test function in $L^{2}(0,T; H^{1}(\mathbb{R}^{2}))$, $\operatorname{supp}\psi \subset\Omega\subset\mathbb{R}^{2}$.

$$\begin{aligned} & \int^{T}_{0} \int_{\mathbb{R}^{2}}\bigl(n^{\alpha}E^{\alpha}-nE\bigr)\psi \,dx\,dt \\ &\quad = \int^{T}_{0} \int_{\Omega}n^{\alpha}\bigl(E^{\alpha}-E\bigr)\psi \,dx \,dt+ \int^{T}_{0} \int _{\Omega}\bigl(n^{\alpha}-n\bigr)E\psi \,dx \,dt. \end{aligned}$$

Since

$$\begin{aligned} & \biggl\vert \int^{T}_{0} \int_{\Omega}n^{\alpha}\bigl(E^{\alpha}-E\bigr)\psi \,dx \,dt \biggr\vert \\ &\quad \leq \bigl\Vert n^{\alpha}\bigr\Vert _{L^{\infty}(0,T; L^{2}(\Omega))} \bigl\Vert E^{\alpha}-E \bigr\Vert _{L^{2}(0,T; L^{4}(\Omega))} \|\psi\|_{L^{2}(0,T; L^{4}(\Omega))}, \end{aligned}$$

we deduce from (12) that

$$ \int^{T}_{0} \int_{\Omega}n^{\alpha}\bigl(E^{\alpha}-E\bigr)\psi \,dx \,dt\rightarrow0 \quad (\alpha \rightarrow0). $$

In fact

$$ \|E\psi\|_{L^{1}(0,T; L^{2}(\mathbb{R}))}\leq\|E\|_{L^{2}(0,T; L^{4}(\mathbb {R}^{2}))} \|\psi\|_{L^{2}(0,T; L^{4}(\mathbb{R}^{2}))}< \infty. $$

Therefore, we deduce from (13) that

$$ \int^{T}_{0} \int_{\Omega}\bigl(n^{\alpha}-n\bigr)E\psi \,dx \,dt \rightarrow0\quad (\alpha \rightarrow0). $$

Thus $n^{\alpha}E^{\alpha}\rightarrow n E$ in $L^{2}(0,T; H^{-1}(\mathbb {R}^{2}))$. So $z=nE$.

We notice that the embedding

$$ H^{1}(\Omega)\rightarrow L^{4}(\Omega) $$

is compact, and for any Banach space *X*, the embedding

$$ L^{\infty}(\mathbb{R}_{+}, X)\rightarrow L^{2}(0,T; X) $$

is continuous. Hence, according to (12), (20) and Lemma 2.1, applied to $B_{0}=H^{1}(\Omega)$, $B=L^{4}(\Omega)$, $B_{1}=H^{-1}(\Omega )$, we obtain that some subsequence of $E^{\alpha}|_{\Omega}$ (also labeled by *α*) converges strongly to $E|_{\Omega}$ in $L^{2}(0,T; L^{4}(\Omega))$. Thus, we can assume that

23 $$ E^{\alpha}\rightarrow E \quad \mbox{strongly in } L^{2}\bigl(0,T; L^{4}_{\mathrm{loc}}(\Omega)\bigr), $$

and thus

24 $$ E^{\alpha}\rightarrow E \quad \mbox{a.e. in } [0,T]\times \mathbb{R}^{2}. $$

Then, using Lemma 2.2, (16) and (24) imply that $w=|E|^{2}$.

Finally, let $\phi\in\mathcal{D}(\mathbb{R}^{2})$, we obtain from (6) that

$$ \bigl(i E_{t}^{\alpha}+ \Delta E^{\alpha}-n^{\alpha}E^{\alpha}+\alpha \bigl\vert E^{\alpha}\bigr\vert ^{p} E^{\alpha}, \phi \bigr)=0. $$

By virtue of $\|E^{\alpha}\|_{L^{\infty}(\mathbb{R}_{+}, H^{1}(\mathbb {R}^{2}))}$ is bounded uniformly in *α*, we can obtain

$$ \bigl\vert \bigl(\alpha \bigl\vert E^{\alpha}\bigr\vert ^{p} E^{\alpha}, \phi \bigr) \bigr\vert \leq|\alpha| \bigl\Vert E^{\alpha}\bigr\Vert ^{p+1}_{L^{2p+2}}\|\phi\| _{L^{2}}\rightarrow0,\quad \alpha\rightarrow0. $$

Thus

$$ i E_{t}^{\alpha}+ \Delta E^{\alpha}-n^{\alpha}E^{\alpha}\rightarrow0\quad \mbox{in } \mathcal{L} \bigl(\mathcal{D}\bigl( \mathbb{R}^{2}\bigr), L^{\infty}(\mathbb {R}_{+}) \bigr). $$

Therefore

$$ i E_{t}+ \Delta E-n E=0, $$

which completes the proof of Theorem 1.1. □

## Strong convergence results

This last result leads us to wondering whether the convergence is better when we take more regular initial data. We shall prove that

$$ F^{\alpha}=E^{\alpha}-E,\qquad u^{\alpha}=n^{\alpha}-n $$

converge strongly in some sense. Taking into account the equations satisfied by $(E^{\alpha}, n^{\alpha})$ and $(E, n)$, we find that $(F^{\alpha}, u^{\alpha})$ must satisfy the system

25 $$\begin{aligned} & i F^{\alpha}_{t}+ \Delta F^{\alpha}-u^{\alpha}F^{\alpha}-u^{\alpha}E-n F^{\alpha}+\alpha \bigl\vert E^{\alpha}\bigr\vert ^{p} E^{\alpha}=0, \end{aligned}$$

26 $$\begin{aligned} & u^{\alpha}_{tt}-\Delta u^{\alpha}= \Delta \bigl( \bigl\vert F^{\alpha}\bigr\vert ^{2}+2\operatorname{Re}\bigl(F^{\alpha}\overline{E} \bigr) \bigr), \end{aligned}$$

with the initial data

27 $$ F^{\alpha}|_{t=0}=0, \qquad u^{\alpha}|_{t=0}=0, \qquad u^{\alpha}_{t}|_{t=0}=0. $$

We introduce $V^{\alpha}(x,t)$ and transform (25)–(27) into the following form:

28 $$\begin{aligned} & i F^{\alpha}_{t}+ \Delta F^{\alpha}-u^{\alpha}F^{\alpha}-u^{\alpha}E-n F^{\alpha}+\alpha \bigl\vert E^{\alpha}\bigr\vert ^{p} E^{\alpha}=0, \end{aligned}$$

29 $$\begin{aligned} & u^{\alpha}_{t}-\Delta V^{\alpha}= 0, \end{aligned}$$

30 $$\begin{aligned} & V^{\alpha}_{t}=u^{\alpha}+ \bigl\vert F^{\alpha}\bigr\vert ^{2}+2\operatorname{Re}\bigl(F^{\alpha}\overline{E} \bigr), \end{aligned}$$

with the initial data

31 $$ F^{\alpha}|_{t=0}=0,\qquad u^{\alpha}|_{t=0}=0, \qquad V^{\alpha}|_{t=0}=0. $$

We know that if $E_{0}(x)\in H^{l+2}(\mathbb{R}^{2})$, $n_{0}(x)\in H^{l+1}(\mathbb{R}^{2})$, $n_{1}(x)\in H^{l}(\mathbb{R}^{2})$, $l\geq1$, and $0< p\leq2$ with $\|E_{0}(x)\|_{L^{2}}$ small, there exists a unique global solution $(E^{\alpha}, n^{\alpha})$ for system (3)–(5) satisfying [17]

$$\begin{aligned} &E^{\alpha}(x,t)\in L^{\infty}_{\mathrm{loc}}\bigl(0,T;H^{l+2} \bigr)\cap W^{1, \infty}\bigl(0, T; H^{l}\bigr),\qquad n^{\alpha}(x,t)\in L^{\infty}_{\mathrm{loc}}\bigl(0,T;H^{l+1} \bigr), \\ &n_{t}^{\alpha}(x,t)\in L^{\infty}_{\mathrm{loc}}\bigl(0, T; H^{l}\bigr)\cap W^{1, \infty}\bigl(0, T; H^{l-1}\bigr). \end{aligned}$$

Moreover, if $E_{0}(x)\in H^{m+2}(\mathbb{R}^{2})$, $n_{0}(x)\in H^{m+1}(\mathbb{R}^{2})$, $n_{1}(x)\in H^{m}(\mathbb{R}^{2})$, $m\geq1$, and $\|E_{0}(x)\|_{L^{2}}$ small, system (1), 2, (5) has a unique global solution $(E, n)$ satisfying [20]

$$\begin{aligned} &E(x,t)\in L^{\infty}_{\mathrm{loc}}\bigl(0,T;H^{m+2}\bigl( \mathbb{R}^{2}\bigr)\bigr),\qquad n(x,t)\in L^{\infty}_{\mathrm{loc}}\bigl(0,T;H^{m+1}\bigl(\mathbb{R}^{2}\bigr) \bigr), \\ &n_{t}\in L^{\infty}_{\mathrm{loc}}\bigl(0,T;H^{m} \bigl(\mathbb{R}^{2}\bigr)\bigr). \end{aligned}$$

The entire proof of Theorem 1.2 is broken down into Lemmas 3.1 and 3.2.

### Lemma 3.1

*Suppose that* $E_{0}\in H^{3}$, $n_{0}\in H^{2}$, $n_{1}\in H^{1}$, $0< p\leq2$, $|\alpha|<1$, *and* $\|E_{0}(x)\|_{L^{2}}$ *small*. *Then there exists some function* $M(t)\in L^{\infty}_{\mathrm{Loc}}(\mathbb{R_{+}})$ *such that*

$$\begin{aligned} \bigl\Vert \nabla F^{\alpha}\bigr\Vert ^{2}_{L^{2}}+ \bigl\Vert F^{\alpha}\bigr\Vert ^{2}_{L^{2}}+ \bigl\Vert \nabla V^{\alpha}\bigr\Vert ^{2}_{L^{2}}+ \bigl\Vert u^{\alpha}\bigr\Vert ^{2}_{L^{2}}\leq M(t) \alpha^{2}. \end{aligned}$$

### Proof

Taking the inner product of (28) and $F^{\alpha}$, we have

32 $$ \bigl(i F^{\alpha}_{t}+ \Delta F^{\alpha}-u^{\alpha}F^{\alpha}-u^{\alpha}E-n F^{\alpha}+\alpha \bigl\vert E^{\alpha}\bigr\vert ^{p} E^{\alpha}, F^{\alpha}\bigr)=0. $$

Since

$$\begin{aligned} &\operatorname{Im}\bigl(i F^{\alpha}_{t}, F^{\alpha}\bigr)= \frac{1}{2}\frac{d}{dt} \bigl\Vert F^{\alpha}\bigr\Vert ^{2}_{L^{2}}, \\ &\operatorname{Im}\bigl( \Delta F^{\alpha}-n^{\alpha}F^{\alpha}, F^{\alpha}\bigr)=0, \\ & \bigl\vert \operatorname{Im}\bigl(-u^{\alpha}E+\alpha \bigl\vert E^{\alpha}\bigr\vert ^{p} E^{\alpha}, F^{\alpha}\bigr) \bigr\vert \\ &\quad \leq \bigl( \Vert E \Vert _{L^{\infty}} \bigl\Vert u^{\alpha}\bigr\Vert _{L^{2}}+|\alpha| \bigl\Vert E^{\alpha}\bigr\Vert ^{p+1}_{L^{2p+2}} \bigr) \bigl\Vert F^{\alpha}\bigr\Vert _{L^{2}} \\ &\quad \leq M(t) \bigl( \bigl\Vert u^{\alpha}\bigr\Vert ^{2}_{L^{2}}+ \bigl\Vert F^{\alpha}\bigr\Vert ^{2}_{L^{2}}+\alpha^{2} \bigr). \end{aligned}$$

From (32), we get

33 $$ \frac{d}{dt} \bigl\Vert F^{\alpha}\bigr\Vert ^{2}_{L^{2}}\leq M(t) \bigl( \bigl\Vert u^{\alpha}\bigr\Vert ^{2}_{L^{2}}+ \bigl\Vert F^{\alpha}\bigr\Vert ^{2}_{L^{2}}+\alpha^{2} \bigr). $$

We deduce the inequality from (33)

34 $$ \bigl\Vert F^{\alpha}\bigr\Vert ^{2}_{L^{2}} \leq M(t) \biggl( \int^{t}_{0} \bigl( \bigl\Vert u^{\alpha}\bigr\Vert ^{2}_{L^{2}}+ \bigl\Vert F^{\alpha}\bigr\Vert ^{2}_{L^{2}} \bigr)\,d\tau +\alpha^{2} \biggr). $$

Taking the inner product of (28) and $F^{\alpha}_{t}$ gives that

35 $$ \bigl(i F^{\alpha}_{t}+ \Delta F^{\alpha}-u^{\alpha}F^{\alpha}-u^{\alpha}E-n F^{\alpha}+\alpha \bigl\vert E^{\alpha}\bigr\vert ^{p} E^{\alpha}, F^{\alpha}_{t} \bigr)=0. $$

Since

$$\begin{aligned}& \operatorname{Re}\bigl(i F^{\alpha}_{t}, F^{\alpha}_{t} \bigr)=0, \\& \operatorname{Re}\bigl(\Delta F^{\alpha}, F^{\alpha}_{t} \bigr)=- \frac{1}{2}\frac {d}{dt} \bigl\Vert \nabla F^{\alpha}\bigr\Vert ^{2}_{L^{2}}, \\& \begin{aligned} \operatorname{Re}\bigl(-u^{\alpha}F^{\alpha}-n F^{\alpha}, F^{\alpha}_{t} \bigr) &=-\frac{1}{2} \int_{\mathbb{R}^{2}} \bigl(u^{\alpha}+n \bigr) \bigl( \bigl\vert F^{\alpha}\bigr\vert ^{2} \bigr)_{t}\,dx \\ &=-\frac{1}{2}\frac{d}{dt} \int_{\mathbb{R}^{2}} \bigl(u^{\alpha}+n \bigr) \bigl\vert F^{\alpha}\bigr\vert ^{2}\,dx +\frac{1}{2} \int_{\mathbb{R}^{2}} \bigl(u^{\alpha}+n \bigr)_{t} \bigl\vert F^{\alpha}\bigr\vert ^{2}\,dx, \end{aligned} \end{aligned}$$

where

$$\begin{aligned} \frac{1}{2} \int_{\mathbb{R}^{2}}u^{\alpha}_{t} \bigl\vert F^{\alpha}\bigr\vert ^{2}\,dx &=\frac{1}{2} \int_{\mathbb{R}^{2}}u^{\alpha}_{t} \bigl(V^{\alpha}_{t}-u^{\alpha}-2 \operatorname{Re}\bigl(F^{\alpha}\overline{E} \bigr) \bigr)\,dx \\ &=\frac{1}{2} \int_{\mathbb{R}^{2}}\Delta V^{\alpha}V^{\alpha}_{t} \,dx-\frac {1}{4}\frac{d}{dt} \bigl\Vert u^{\alpha}\bigr\Vert ^{2}_{L^{2}} -\frac{d}{dt} \int_{\mathbb{R}^{2}}u^{\alpha} \operatorname{Re}\bigl(F^{\alpha}\overline {E} \bigr)\,dx \\ &\quad {} + \int_{\mathbb{R}^{2}}u^{\alpha} \operatorname{Re}\bigl(F^{\alpha}\overline{E} \bigr)_{t}\,dx \\ &=-\frac{1}{4}\frac{d}{dt} \biggl( \bigl\Vert \nabla V^{\alpha}\bigr\Vert ^{2}_{L^{2}}+ \bigl\Vert u^{\alpha}\bigr\Vert ^{2}_{L^{2}}+4 \int_{\mathbb{R}^{2}}u^{\alpha} \operatorname{Re}\bigl(F^{\alpha}\overline{E} \bigr)\,dx \biggr) \\ &\quad {} + \int_{\mathbb{R}^{2}}u^{\alpha} \operatorname{Re}\bigl(F^{\alpha}\overline{E} \bigr)_{t}\,dx. \end{aligned}$$

From (35), we obtain

36 $$\begin{aligned} &\frac{d}{dt} \biggl(\frac{1}{2} \bigl\Vert \nabla F^{\alpha}\bigr\Vert ^{2}_{L^{2}}+\frac{1}{4} \bigl\Vert \nabla V^{\alpha}\bigr\Vert ^{2}_{L^{2}}+\frac {1}{4} \bigl\Vert u^{\alpha}\bigr\Vert ^{2}_{L^{2}} + \frac{1}{2} \int_{\mathbb{R}^{2}} \bigl(u^{\alpha}+n \bigr) \bigl\vert F^{\alpha}\bigr\vert ^{2}\,dx \biggr) \\ &\qquad {} +\frac{d}{dt} \int_{\mathbb{R}^{2}}u^{\alpha} \operatorname{Re}\bigl(F^{\alpha}\overline {E} \bigr)\,dx \\ &\quad = \int_{\mathbb{R}^{2}}u^{\alpha} \operatorname{Re}\bigl(F^{\alpha}\overline{E}_{t} \bigr)\,dx+\frac{1}{2} \int_{\mathbb{R}^{2}}n_{t} \bigl\vert F^{\alpha}\bigr\vert ^{2}\,dx +\alpha \operatorname{Re}\int_{\mathbb{R}^{2}} \bigl\vert E^{\alpha}\bigr\vert ^{p} E^{\alpha}\overline {F^{\alpha}_{t}} \,dx. \end{aligned}$$

First, we study the right-hand side of (36).

$$\begin{aligned}& \begin{aligned} \biggl\vert \int_{\mathbb{R}^{2}}u^{\alpha} \operatorname{Re}\bigl(F^{\alpha}\overline{E}_{t} \bigr)\,dx \biggr\vert &\leq \Vert E_{t} \Vert _{L^{4}} \bigl\Vert u^{\alpha}\bigr\Vert _{L^{2}} \bigl\Vert F^{\alpha}\bigr\Vert _{L^{4}} \\ &\leq M(t) \bigl\Vert u^{\alpha}\bigr\Vert _{L^{2}} \bigl\Vert \nabla F^{\alpha}\bigr\Vert ^{\frac{1}{2}}_{L^{2}} \bigl\Vert F^{\alpha}\bigr\Vert ^{\frac{1}{2}}_{L^{2}} \\ &\leq M(t) \bigl( \bigl\Vert u^{\alpha}\bigr\Vert ^{2}_{L^{2}}+ \bigl\Vert \nabla F^{\alpha}\bigr\Vert ^{2}_{L^{2}}+ \bigl\Vert F^{\alpha}\bigr\Vert ^{2}_{L^{2}} \bigr), \end{aligned} \\& \begin{aligned} \biggl\vert \frac{1}{2} \int_{\mathbb{R}^{2}}n_{t} \bigl\vert F^{\alpha}\bigr\vert ^{2}\,dx \biggr\vert &\leq\frac{1}{2} \Vert n_{t} \Vert _{L^{2}} \bigl\Vert F^{\alpha}\bigr\Vert ^{2}_{L^{4}} \\ &\leq M(t) \bigl\Vert \nabla F^{\alpha}\bigr\Vert _{L^{2}} \bigl\Vert F^{\alpha}\bigr\Vert _{L^{2}} \\ &\leq M(t) \bigl( \bigl\Vert \nabla F^{\alpha}\bigr\Vert ^{2}_{L^{2}}+ \bigl\Vert F^{\alpha}\bigr\Vert ^{2}_{L^{2}} \bigr), \end{aligned} \\& \begin{aligned} & \biggl\vert \alpha \operatorname{Re}\int_{\mathbb{R}^{2}} \bigl\vert E^{\alpha}\bigr\vert ^{p} E^{\alpha}\overline{F}^{\alpha}_{t}\,dx \biggr\vert \\ &\quad =|\alpha| \biggl\vert \operatorname{Re}\int_{\mathbb{R}^{2}} i \bigl\vert E^{\alpha}\bigr\vert ^{p} E^{\alpha}\bigl(\Delta\overline{F^{\alpha}}-u^{\alpha}\overline{E^{\alpha}}-n \overline{F^{\alpha}}-\alpha \bigl\vert E^{\alpha}\bigr\vert ^{p} \overline{E^{\alpha}} \bigr) \,dx \biggr\vert \\ &\quad \leq C |\alpha| \bigl( \bigl\Vert E^{\alpha}\bigr\Vert ^{p}_{L^{\infty}} \bigl\Vert \nabla E^{\alpha}\bigr\Vert _{L^{2}} \bigl\Vert \nabla F^{\alpha}\bigr\Vert _{L^{2}}+ \bigl\Vert E^{\alpha}\bigr\Vert ^{p+1}_{L^{\infty}} \bigl\Vert E^{\alpha}\bigr\Vert _{L^{2}} \bigl\Vert u^{\alpha}\bigr\Vert _{L^{2}} \bigr) \\ &\qquad {} + |\alpha| \bigl( \Vert n \Vert _{L^{\infty}} \bigl\Vert E^{\alpha}\bigr\Vert ^{p+1}_{L^{2p+2}} \bigl\Vert F^{\alpha}\bigr\Vert _{L^{2}} +|\alpha| \bigl\Vert E^{\alpha}\bigr\Vert ^{2p+2}_{L^{2p+2}} \bigr) \\ &\quad \leq M(t) \bigl( \bigl\Vert \nabla F^{\alpha}\bigr\Vert ^{2}_{L^{2}}+ \bigl\Vert u^{\alpha}\bigr\Vert ^{2}_{L^{2}}+ \bigl\Vert F^{\alpha}\bigr\Vert ^{2}_{L^{2}}+\alpha^{2} \bigr). \end{aligned} \end{aligned}$$

Thus, the right-hand side of (36) is smaller than

$$ M(t) \bigl( \bigl\Vert \nabla F^{\alpha}\bigr\Vert ^{2}_{L^{2}}+ \bigl\Vert F^{\alpha}\bigr\Vert ^{2}_{L^{2}}+ \bigl\Vert u^{\alpha}\bigr\Vert ^{2}_{L^{2}}+\alpha^{2} \bigr). $$

We now study the left-hand side of (36).

$$\begin{aligned}& \biggl\vert \frac{1}{2} \int_{\mathbb{R}^{2}} \bigl(u^{\alpha}+n \bigr) \bigl\vert F^{\alpha}\bigr\vert ^{2}\,dx \biggr\vert \leq \frac{1}{2} \bigl\Vert n^{\alpha}\bigr\Vert _{L^{\infty}} \bigl\Vert F^{\alpha}\bigr\Vert ^{2}_{L^{2}} \leq M(t) \bigl\Vert F^{\alpha}\bigr\Vert ^{2}_{L^{2}}, \\& \begin{aligned} \biggl\vert \int_{\mathbb{R}^{2}}u^{\alpha} \operatorname{Re}\bigl(F^{\alpha}\overline{E} \bigr)\,dx \biggr\vert &\leq\|E\|_{L^{\infty}} \bigl\Vert u^{\alpha}\bigr\Vert _{L^{2}} \bigl\Vert F^{\alpha}\bigr\Vert _{L^{2}} \\ &\leq M(t) \bigl\Vert F^{\alpha}\bigr\Vert ^{2}_{L^{2}}+ \frac{1}{8} \bigl\Vert u^{\alpha}\bigr\Vert ^{2}_{L^{2}}. \end{aligned} \end{aligned}$$

Thus, taking into account the initial data, we deduce from (36) the inequality

37 $$\begin{aligned} &\frac{1}{2} \bigl\Vert \nabla F^{\alpha}\bigr\Vert ^{2}_{L^{2}}+\frac{1}{4} \bigl\Vert \nabla V^{\alpha}\bigr\Vert ^{2}_{L^{2}}+\frac{1}{8} \bigl\Vert u^{\alpha}\bigr\Vert ^{2}_{L^{2}} \\ &\quad \leq M(t) \biggl( \bigl\Vert F^{\alpha}\bigr\Vert ^{2}_{L^{2}} + \int^{t}_{0} \bigl( \bigl\Vert \nabla F^{\alpha}\bigr\Vert ^{2}_{L^{2}}+ \bigl\Vert F^{\alpha}\bigr\Vert ^{2}_{L^{2}}+ \bigl\Vert u^{\alpha}\bigr\Vert ^{2}_{L^{2}} \bigr)\,d\tau+\alpha ^{2} \biggr). \end{aligned}$$

By combining inequalities (34) and (37), we obtain the estimate

$$\begin{aligned} & \bigl\Vert \nabla F^{\alpha}\bigr\Vert ^{2}_{L^{2}}+ \bigl\Vert F^{\alpha}\bigr\Vert ^{2}_{L^{2}}+ \bigl\Vert \nabla V^{\alpha}\bigr\Vert ^{2}_{L^{2}}+ \bigl\Vert u^{\alpha}\bigr\Vert ^{2}_{L^{2}} \\ &\quad \leq M(t) \biggl( \int^{t}_{0} \bigl( \bigl\Vert \nabla F^{\alpha}\bigr\Vert ^{2}_{L^{2}}+ \bigl\Vert F^{\alpha}\bigr\Vert ^{2}_{L^{2}}+ \bigl\Vert \nabla V^{\alpha}\bigr\Vert ^{2}_{L^{2}}+ \bigl\Vert u^{\alpha}\bigr\Vert ^{2}_{L^{2}} \bigr)\,d\tau+ \alpha^{2} \biggr), \end{aligned}$$

which immediately yields

$$ \bigl\Vert \nabla F^{\alpha}\bigr\Vert ^{2}_{L^{2}}+ \bigl\Vert F^{\alpha}\bigr\Vert ^{2}_{L^{2}}+ \bigl\Vert \nabla V^{\alpha}\bigr\Vert ^{2}_{L^{2}}+ \bigl\Vert u^{\alpha}\bigr\Vert ^{2}_{L^{2}}\leq M(t) \alpha^{2}. $$

 □

### Lemma 3.2

*Suppose that* $E_{0}\in H^{m+2}$, $n_{0}\in H^{m+1}$, $n_{1}\in H^{m}$ ($m\geq 1$) , $0< p\leq2$, $|\alpha|<1$, *and* $\|E_{0}(x)\|_{L^{2}}$ *small*. *Then there exists some function* $M(t)\in L^{\infty}_{\mathrm{Loc}}(\mathbb{R_{+}})$ *such that*

38 $$ \bigl\Vert D^{m}F^{\alpha}\bigr\Vert ^{2}_{L^{2}}+ \bigl\Vert D^{m-1}F^{\alpha}\bigr\Vert ^{2}_{L^{2}}+ \bigl\Vert D^{m}V^{\alpha}\bigr\Vert ^{2}_{L^{2}}+ \bigl\Vert D^{m-1}u^{\alpha}\bigr\Vert ^{2}_{L^{2}}\leq M(t)\alpha^{2}. $$

### Proof

Lemma 3.2 is true when $m=1$ (Lemma 3.1). Suppose that Lemma 3.2 is true when $m=k$ ($k\geq1$), i.e.,

$$ \bigl\Vert D^{k}F^{\alpha}\bigr\Vert ^{2}_{L^{2}}+ \bigl\Vert D^{k-1}F^{\alpha}\bigr\Vert ^{2}_{L^{2}}+ \bigl\Vert D^{k}V^{\alpha}\bigr\Vert ^{2}_{L^{2}}+ \bigl\Vert D^{k-1}u^{\alpha}\bigr\Vert ^{2}_{L^{2}} \leq M(t)\alpha^{2}. $$

We shall prove that estimate (38) is true when $m=k+1$.

Taking the inner product of (28) and $(-1)^{k+1}D^{2k}F^{\alpha}_{t}$ results in

39 $$ \bigl(i F^{\alpha}_{t}+ \Delta F^{\alpha}-u^{\alpha}F^{\alpha}-u^{\alpha}E-n F^{\alpha}+\alpha \bigl\vert E^{\alpha}\bigr\vert ^{p} E^{\alpha}, (-1)^{k+1}D^{2k}F^{\alpha}_{t} \bigr)=0. $$

Since

$$\begin{aligned}& \operatorname{Re}\bigl(i F^{\alpha}_{t}, (-1)^{k+1}D^{2k}F^{\alpha}_{t} \bigr)=0, \\& \operatorname{Re}\bigl( \Delta F^{\alpha}, (-1)^{k+1}D^{2k}F^{\alpha}_{t} \bigr)=\frac {1}{2}\frac{d}{dt} \bigl\Vert D^{k+1}F^{\alpha}\bigr\Vert ^{2}_{L^{2}}. \\& \begin{aligned} \operatorname{Re}\bigl(-u^{\alpha}E^{\alpha}, (-1)^{k+1}D^{2k}F^{\alpha}_{t} \bigr) &=-\operatorname{Re}\bigl(D^{k-1} \bigl(u^{\alpha}E^{\alpha}\bigr), D^{k+1}F^{\alpha}_{t} \bigr) \\ &=-\frac{d}{dt}\operatorname{Re}\bigl(D^{k-1} \bigl(u^{\alpha}E^{\alpha}\bigr), D^{k+1}F^{\alpha}\bigr) \\ &\quad {} +\operatorname{Re}\bigl(D^{k-1} \bigl(u^{\alpha}E^{\alpha}\bigr)_{t}, D^{k+1}F^{\alpha}\bigr), \end{aligned} \\& \operatorname{Re}\bigl(-n F^{\alpha}, (-1)^{k+1}D^{2k}F^{\alpha}_{t} \bigr) \\& \quad =\operatorname{Re}\bigl(D^{k} \bigl(n F^{\alpha}\bigr), D^{k}F^{\alpha}_{t} \bigr) \\& \quad =\frac{1}{2} \int_{\mathbb{R}^{2}}n \bigl( \bigl\vert D^{k}F^{\alpha}\bigr\vert ^{2} \bigr)_{t}\,dx+\operatorname{Re}\Biggl(\sum ^{k}_{j=1}C^{j}_{k} D^{j} n D^{k-j}F^{\alpha}, D^{k}F^{\alpha}_{t} \Biggr) \\& \quad =\frac{1}{2}\frac{d}{dt} \int_{\mathbb{R}^{2}}n \bigl\vert D^{k}F^{\alpha}\bigr\vert ^{2}\,dx-\frac{1}{2} \int_{\mathbb{R}^{2}}n_{t} \bigl\vert D^{k}F^{\alpha}\bigr\vert ^{2}\,dx \\& \qquad {} +\operatorname{Re}\Biggl(\sum^{k}_{j=1}C^{j}_{k} D^{j} n D^{k-j}F^{\alpha}, D^{k}F^{\alpha}_{t} \Biggr), \\& \begin{aligned} \operatorname{Re}\bigl(\alpha \bigl\vert E^{\alpha}\bigr\vert ^{p}E^{\alpha}, (-1)^{k+1}D^{2k}F^{\alpha}_{t} \bigr)&=\alpha \operatorname{Re}\bigl(D^{k-1} \bigl( \bigl\vert E^{\alpha}\bigr\vert ^{p}E^{\alpha}\bigr), D^{k+1}F^{\alpha}_{t} \bigr) \\ &=\alpha\frac{d}{dt} \operatorname{Re}\bigl(D^{k-1} \bigl( \bigl\vert E^{\alpha}\bigr\vert ^{p}E^{\alpha}\bigr), D^{k+1}F^{\alpha}\bigr) \\ &\quad {} -\alpha \operatorname{Re}\bigl(D^{k-1} \bigl( \bigl\vert E^{\alpha}\bigr\vert ^{p}E^{\alpha}\bigr)_{t}, D^{k+1}F^{\alpha}\bigr). \end{aligned} \end{aligned}$$

Thus, from (39), we get

40 $$\begin{aligned} &\frac{d}{dt} \biggl(\frac{1}{2} \bigl\Vert D^{k+1}F^{\alpha}\bigr\Vert ^{2}_{L^{2}}-\operatorname{Re}\bigl(D^{k-1} \bigl(u^{\alpha}E^{\alpha}\bigr), D^{k+1}F^{\alpha}\bigr) +\frac{1}{2} \int_{\mathbb{R}^{2}}n \bigl\vert D^{k}F^{\alpha}\bigr\vert ^{2}\,dx \biggr) \\ &\qquad {} +\alpha\frac{d}{dt}\operatorname{Re}\bigl(D^{k-1} \bigl( \bigl\vert E^{\alpha}\bigr\vert ^{p}E^{\alpha}\bigr), D^{k+1}F^{\alpha}\bigr) \\ &\quad =\frac{1}{2} \int_{\mathbb{R}^{2}}n_{t} \bigl\vert D^{k}F^{\alpha}\bigr\vert ^{2}\,dx-\operatorname{Re}\Biggl(\sum^{k}_{j=1}C^{j}_{k} D^{j} n D^{k-j}F^{\alpha}, D^{k}F^{\alpha}_{t} \Biggr) \\ &\qquad {} -\operatorname{Re}\bigl(D^{k-1} \bigl(u^{\alpha}E^{\alpha}\bigr)_{t}, D^{k+1}F^{\alpha}\bigr)+\alpha \operatorname{Re}\bigl(D^{k-1} \bigl( \bigl\vert E^{\alpha}\bigr\vert ^{p}E^{\alpha}\bigr)_{t}, D^{k+1}F^{\alpha}\bigr). \end{aligned}$$

Since

$$\begin{aligned}& \begin{aligned} \biggl\vert \frac{1}{2} \int_{\mathbb{R}^{2}}n_{t} \bigl\vert D^{k}F^{\alpha}\bigr\vert ^{2}\,dx \biggr\vert &\leq\frac{1}{2} \Vert n_{t} \Vert _{L^{2}} \bigl\Vert D^{k}F^{\alpha}\bigr\Vert ^{2}_{L^{4}} \\ &\leq M(t) \bigl\Vert D^{k+1}F^{\alpha}\bigr\Vert _{L^{2}} \bigl\Vert D^{k}F^{\alpha}\bigr\Vert _{L^{2}} \\ &\leq M(t) \bigl( \bigl\Vert D^{k+1}F^{\alpha}\bigr\Vert ^{2}_{L^{2}}+\alpha^{2} \bigr), \end{aligned} \\& \Biggl\vert \operatorname{Re}\Biggl(\sum^{k}_{j=1}C^{j}_{k} D^{j} n D^{k-j}F^{\alpha}, D^{k}F^{\alpha}_{t} \Biggr) \Biggr\vert \\& \quad = \Biggl\vert \operatorname{Re}\Biggl(\sum^{k}_{j=1}C^{j}_{k} D^{j} n D^{k-j}F^{\alpha}, -iD^{k} \bigl( \bar{F}^{\alpha}_{xx}-u^{\alpha}\bar{E}^{\alpha}-n \bar {F}^{\alpha}-\alpha \bigl\vert E^{\alpha}\bigr\vert ^{p} \bar{E}^{\alpha}\bigr) \Biggr) \Biggr\vert \\& \quad \leq M(t) \bigl( \bigl\Vert D^{k+1}F^{\alpha}\bigr\Vert ^{2}_{L^{2}}+ \bigl\Vert D^{k}u^{\alpha}\bigr\Vert ^{2}_{L^{2}}+\alpha^{2} \bigr), \\& \begin{aligned} \bigl\vert \operatorname{Re}\bigl(D^{k-1} \bigl(u^{\alpha}E^{\alpha}\bigr)_{t}, D^{k+1}F^{\alpha}\bigr) \bigr\vert &= \bigl\vert \operatorname{Re}\bigl(D^{k-1} \bigl(V^{\alpha}_{xx} E^{\alpha}+u^{\alpha}E^{\alpha}_{t} \bigr), D^{k+1}F^{\alpha}\bigr) \bigr\vert \\ &\leq M(t) \bigl( \bigl\Vert D^{k+1}V^{\alpha}\bigr\Vert ^{2}_{L^{2}}+ \bigl\Vert D^{k+1}F^{\alpha}\bigr\Vert ^{2}_{L^{2}}+\alpha^{2} \bigr), \end{aligned} \\& \bigl\vert \alpha \operatorname{Re}\bigl(D^{k-1} \bigl( \bigl\vert E^{\alpha}\bigr\vert ^{p}E^{\alpha}\bigr)_{t}, D^{k+1}F^{\alpha}\bigr) \bigr\vert \leq M(t) \bigl( \bigl\Vert D^{k+1}F^{\alpha}\bigr\Vert ^{2}_{L^{2}}+\alpha^{2} \bigr). \end{aligned}$$

Thus, the right-hand side of (40) is smaller than

$$ M(t) \bigl( \bigl\Vert D^{k+1}F^{\alpha}\bigr\Vert ^{2}_{L^{2}}+ \bigl\Vert D^{k+1}V^{\alpha}\bigr\Vert ^{2}_{L^{2}}+ \bigl\Vert D^{k}u^{\alpha}\bigr\Vert ^{2}_{L^{2}}+\alpha^{2} \bigr). $$

We now study the left-hand side of (40).

$$\begin{aligned}& \bigl\vert \operatorname{Re}\bigl(D^{k-1} \bigl(u^{\alpha}E^{\alpha}\bigr), D^{k+1}F^{\alpha}\bigr) \bigr\vert \leq M(t) \alpha^{2}+\frac{1}{8} \bigl\Vert D^{k+1}F^{\alpha}\bigr\Vert ^{2}_{L^{2}}, \\& \biggl\vert \frac{1}{2} \int_{\mathbb{R}^{2}}n \bigl\vert D^{k}F^{\alpha}\bigr\vert ^{2}\,dx \biggr\vert \leq\frac{1}{2} \Vert n \Vert _{L^{\infty}} \bigl\Vert D^{k}F^{\alpha}\bigr\Vert ^{2}_{L^{2}}\leq M(t) \alpha^{2}, \\& \bigl\vert \alpha \operatorname{Re}\bigl(D^{k-1} \bigl( \bigl\vert E^{\alpha}\bigr\vert ^{p}E^{\alpha}\bigr), D^{k+1}F^{\alpha}\bigr) \bigr\vert \leq M(t) \alpha^{2}+\frac{1}{8} \bigl\Vert D^{k+1}F^{\alpha}\bigr\Vert ^{2}_{L^{2}}. \end{aligned}$$

Thus, taking into account the initial data, we deduce from (40) the following inequality:

41 $$ \bigl\Vert D^{k+1}F^{\alpha}\bigr\Vert ^{2}_{L^{2}}\leq M(t) \biggl( \int^{t}_{0} \bigl( \bigl\Vert D^{k+1}F^{\alpha}\bigr\Vert ^{2}_{L^{2}}+ \bigl\Vert D^{k+1}V^{\alpha}\bigr\Vert ^{2}_{L^{2}}+ \bigl\Vert D^{k}u^{\alpha}\bigr\Vert ^{2}_{L^{2}} \bigr)\,d\tau+\alpha^{2} \biggr). $$

Taking the inner product of (30) and $(-1)^{k+1}D^{2k+2} V^{\alpha}$ leads to

42 $$\begin{aligned} & \bigl(V^{\alpha}_{t}, (-1)^{k+1}D^{2k+2} V^{\alpha}\bigr) \\ &\quad = \bigl(u^{\alpha}+ \bigl\vert F^{\alpha}\bigr\vert ^{2}+2\operatorname{Re}\bigl(F^{\alpha}\overline{E} \bigr), (-1)^{k+1}D^{2k+2} V^{\alpha}\bigr). \end{aligned}$$

Since

$$\begin{aligned}& \bigl(V^{\alpha}_{t}, (-1)^{k+1}D^{2k+2} V^{\alpha}\bigr)=\frac{1}{2}\frac {d}{dt} \bigl\Vert D^{k+1}V^{\alpha}\bigr\Vert ^{2}_{L^{2}}, \\& \bigl(u^{\alpha}, (-1)^{k+1}D^{2k+2} V^{\alpha}\bigr)= \bigl(u^{\alpha}, (-1)^{k+1}D^{2k} u^{\alpha}_{t} \bigr)=-\frac{1}{2}\frac{d}{dt} \bigl\Vert D^{k}u^{\alpha}\bigr\Vert ^{2}_{L^{2}}, \\& \begin{aligned} \bigl\vert \bigl( \bigl\vert F^{\alpha}\bigr\vert ^{2}, (-1)^{k+1}D^{2k+2} V^{\alpha}\bigr) \bigr\vert &= \bigl\vert \bigl(D^{k+1} \bigl( \bigl\vert F^{\alpha}\bigr\vert ^{2} \bigr), D^{k+1} V^{\alpha}\bigr) \bigr\vert \\ &\leq M(t) \bigl( \bigl\Vert D^{k+1}F^{\alpha}\bigr\Vert ^{2}_{L^{2}}+ \bigl\Vert D^{k+1}V^{\alpha}\bigr\Vert ^{2}_{L^{2}}+\alpha^{2} \bigr), \end{aligned} \\& \bigl\vert \bigl(2\operatorname{Re}\bigl(F^{\alpha}\overline{E} \bigr), (-1)^{k+1}D^{2k+2} V^{\alpha}\bigr) \bigr\vert \\& \quad =2 \bigl\vert \operatorname{Re}\bigl( D^{k+1} \bigl( F^{\alpha}\overline{E} \bigr), D^{k+1} V^{\alpha}\bigr) \bigr\vert \\& \quad \leq M(t) \bigl( \bigl\Vert D^{k+1}F^{\alpha}\bigr\Vert ^{2}_{L^{2}}+ \bigl\Vert D^{k+1}V^{\alpha}\bigr\Vert ^{2}_{L^{2}}+\alpha^{2} \bigr). \end{aligned}$$

Thus, from (42), we get

43 $$\begin{aligned} &\frac{d}{dt} \bigl( \bigl\Vert D^{k+1}V^{\alpha}\bigr\Vert ^{2}_{L^{2}}+ \bigl\Vert D^{k}u^{\alpha}\bigr\Vert ^{2}_{L^{2}} \bigr) \\ &\quad \leq M(t) \bigl( \bigl\Vert D^{k+1}F^{\alpha}\bigr\Vert ^{2}_{L^{2}}+ \bigl\Vert D^{k+1}V^{\alpha}\bigr\Vert ^{2}_{L^{2}}+\alpha^{2} \bigr). \end{aligned}$$

Thus, taking into account the initial data, we deduce from (43) the following inequality:

44 $$ \bigl\Vert D^{k+1}V^{\alpha}\bigr\Vert ^{2}_{L^{2}}+ \bigl\Vert D^{k}u^{\alpha}\bigr\Vert ^{2}_{L^{2}} \leq M(t) \biggl( \int^{t}_{0} \bigl( \bigl\Vert D^{k+1}F^{\alpha}\bigr\Vert ^{2}_{L^{2}}+ \bigl\Vert D^{k+1}V^{\alpha}\bigr\Vert ^{2}_{L^{2}} \bigr)\,d\tau+\alpha ^{2} \biggr). $$

By combining (41) with (44), we get

$$\begin{aligned} & \bigl\Vert D^{k+1}F^{\alpha}\bigr\Vert ^{2}_{L^{2}}+ \bigl\Vert D^{k+1}V^{\alpha}\bigr\Vert ^{2}_{L^{2}}+ \bigl\Vert D^{k}u^{\alpha}\bigr\Vert ^{2}_{L^{2}} \\ &\quad \leq M(t) \biggl( \int^{t}_{0} \bigl( \bigl\Vert D^{k+1}F^{\alpha}\bigr\Vert ^{2}_{L^{2}}+ \bigl\Vert D^{k+1}V^{\alpha}\bigr\Vert ^{2}_{L^{2}}+ \bigl\Vert D^{k}u^{\alpha}\bigr\Vert ^{2}_{L^{2}} \bigr)\,d\tau +\alpha^{2} \biggr), \end{aligned}$$

which immediately yields

$$ \bigl\Vert D^{k+1}F^{\alpha}\bigr\Vert ^{2}_{L^{2}}+ \bigl\Vert D^{k+1}V^{\alpha}\bigr\Vert ^{2}_{L^{2}}+ \bigl\Vert D^{k}u^{\alpha}\bigr\Vert ^{2}_{L^{2}} \leq M(t)\alpha^{2}. $$

Lemma 3.2 is proved, and the result of Theorem 1.2 is obvious. □

## Results and discussion

One can regard the existence and uniqueness of the global smooth solution for the initial value problem of generalized Zakharov equations (3)–(5) in dimension three and study the asymptotic behavior of system (3)–(5) in dimension three when *α* goes to zero.

## Conclusions

We consider the initial value problem for the generalized Zakharov equations (3)–(5) which model the propagation of Langmuir waves in plasmas. For suitable initial data, solutions are shown to exist for a time interval independent of *α*. For such data, solutions of (3)–(5) converge as $\alpha\to0$ to the solution of the classic Zakharov equations (1)–(2) with (5).
